# An Enhanced Differential Evolution Algorithm Based on Multiple Mutation Strategies

**DOI:** 10.1155/2015/285730

**Published:** 2015-11-01

**Authors:** Wan-li Xiang, Xue-lei Meng, Mei-qing An, Yin-zhen Li, Ming-xia Gao

**Affiliations:** School of Traffic & Transportation, Lanzhou Jiaotong University, Lanzhou, Gansu 730070, China

## Abstract

Differential evolution algorithm is a simple yet efficient metaheuristic for global optimization over continuous spaces. However, there is a shortcoming of premature convergence in standard DE, especially in DE/best/1/bin. In order to take advantage of direction guidance information of the best individual of DE/best/1/bin and avoid getting into local trap, based on multiple mutation strategies, an enhanced differential evolution algorithm, named EDE, is proposed in this paper. In the EDE algorithm, an initialization technique, opposition-based learning initialization for improving the initial solution quality, and a new combined mutation strategy composed of DE/current/1/bin together with DE/*p*best/bin/1 for the sake of accelerating standard DE and preventing DE from clustering around the global best individual, as well as a perturbation scheme for further avoiding premature convergence, are integrated. In addition, we also introduce two linear time-varying functions, which are used to decide which solution search equation is chosen at the phases of mutation and perturbation, respectively. Experimental results tested on twenty-five benchmark functions show that EDE is far better than the standard DE. In further comparisons, EDE is compared with other five state-of-the-art approaches and related results show that EDE is still superior to or at least equal to these methods on most of benchmark functions.

## 1. Introduction

Optimization problems are ubiquitous in the various areas including production, life, and scientific community. These optimization problems are usually nonlinear and nondifferentiable. Particularly, the number of their local optima may increase exponentially with the problem size. Thus, evolutionary algorithms (EAs) only needing the value information of objective functions have many more advantages and have drawn more and more attention of many researchers all over the world. In this way, a lot of researchers have developed a great number of evolutionary algorithms, such as genetic algorithms (GAs), particle swarm optimization (PSO), ant colony optimization (ACO), and differential evolution (DE) algorithm. Among them, differential evolution is one of the most powerful stochastic real-parameter optimization algorithms [1]. It was originally developed by Storn and Price [2, 3] in 1995.

Due to its simple implementation, few control parameters, and fast convergence, DE has been widely and successfully applied in function optimization problems [2–26], constrained optimization problems [27–29], multiobjective optimization problems [30], scheduling [31–33], and others [34–39].

According to the aforementioned statements, it can be seen that DE has been very successful in solving various optimization problems. As far as the type of optimization problems is concerned, more researches mainly focus on continuous function optimization. However, the convergence precision and convergence speed over function optimization are still to be improved. That is, the exploration ability and exploitation ability of DE cannot be well balanced. To overcome the shortage of imbalance of the two abilities, more and more researchers have developed a large number of DE variants. For example, Noman and Iba [11] proposed a kind of accelerated differential evolution by incorporating an adaptive local search technique. Rahnamayan et al. [13] proposed an opposition-based differential evolution (ODE for short), in which a novel opposition-based learning (OBL) technique and a generation-jumping scheme are employed. Qin et al. [14] proposed a self-adaptive differential evolution algorithm, called SaDE, in which both trial vector generation strategies and their associated parameter values are dynamically self-adapted during the process of producing promising solutions. Zhang and Sanderson [15] proposed a novel differential evolution referred to as JADE, in which a novel self-adaptive parameters scheme and a new mutation strategy with optional archive are proposed. And these improvements made JADE achieve a very fast convergence speed and high-quality solutions. Subsequently, Gong et al. [22, 23] proposed a few enhanced DE versions based on JADE by introducing adaptive strategy selection schemes or control parameters adaption mechanisms. In summary, all these state-of-the-art DE variants have achieved better convergence performance than the traditional DE.

Unfortunately, up to now, there exists no specific DE version to substantially achieve the best solution for all optimization problems because the exploration and the exploitation often mutually contradict in reality. Hence, searching for better approaches is very necessary. In order to solve continuous optimization problems more efficiently, an enhanced differential evolution algorithm based on multiple mutation strategies, called EDE for short, is presented in this paper.

The structure of the paper is organized as follows. The standard differential evolution algorithm is described briefly in Section 2. In Section 3, an enhanced differential evolution algorithm is presented and described in detail. Subsequently, Section 4 employs a set of benchmark functions to comprehensively investigate the performance of the proposed algorithm through experimental results of these functions and comparisons with other well-known evolutionary algorithms. Finally, conclusions and further study directions are given in Section 5.

## 2. Differential Evolution Algorithm

Differential evolution algorithm was first proposed by Storn and Price [2, 3]. Like other evolutionary algorithms, an initialization phase is its first task. In addition, it also consists of three major operations: mutation, crossover, and selection. Meanwhile, there exist a few mutation strategies proposed in the work [3]. In order to distinguish the different DE versions with various mutation strategies or different crossover schemes, the famous notation DE/*x*/*y*/*z* was introduced in the literature [3], where *x* represents the vector to be mutated, *y* is the number of differential vectors used, and *z* denotes the crossover scheme employed. DE/rand/1/bin was applied most commonly and it was also usually considered as the canonical DE version. To be specific, the canonical DE version can be described as follows.

### 2.1. Initialization

At the first step, a population of NP individuals is generated randomly by the following form:

(1) $$\begin{array}{c} x_{ij}=x_{j}^{min}+\left(x_{j}^{max}-x_{j}^{min}\right)\cdot \mathrm{rand}\left(0,1\right), \end{array}$$

where *i* = 1,2,…, NP, *j* = 1,2,…, *D*; *x*_*j*_^min^ and *x*_*j*_^max^ are the lower bound and upper bounds of the parameter *j*, respectively. Then, the cost function of each solution is evaluated.

### 2.2. Mutation

Mutation strategy is very important in DE. At the step, a mutant vector *v*_*i*_ is generated by the following formula for each *D*-dimensional target vector *x*_*i*_:

(2) $$\begin{array}{c} v_{i}=x_{a}+F\cdot \left(x_{b}-x_{c}\right), \end{array}$$

where *i* = 1,2,…, NP, *a*, *b*, *c* ∈ {1,2,…, NP} are mutually different random integer number, and they are such that *a* ≠ *b* ≠ *c* ≠ *i*. The mutation scale factor *F* is a real and constant factor ∈[0,2] which controls the amplification of the differential variation (*x*_*b*_ − *x*_*c*_) [3].

### 2.3. Crossover

In order to exchange information between a mutant vector *v*_*i*_ and the current target vector *x*_*i*_, crossover operation is introduced. At this time, a trial vector *u*_*i*_ = (*u*_*i*1_, *u*_*i*2_,…, *u*_*iD*_) is produced by the following form:

(3) $$\begin{array}{c} u_{ij}=\left\{\begin{array}{cc} v_{ij}, & \text{if }\mathrm{rand}{\left[0,1\right]}_{j}\leq \text{Cr}\vee j==j_{rand},\\ x_{ij}, & \text{otherwise}, \end{array}\right. \end{array}$$

where *j* = 1,2,…, *D*, rand⁡[0,1]_*j*_ is a random real number between [0,1], and *j*_rand_ ∈ {1,2,…, *D*} is a randomly chosen index, which ensures that the trial vector *u*_*i*_ obtains at least one parameter from the mutant vector *v*_*i*_. Crossover rate Cr is a predefined constant within the range [0,1] and it controls the fraction of parameter values copied from the mutant vector.

### 2.4. Selection

After crossover operation, the trial vector *u*_*i*_ is compared to the target vector *x*_*i*_ through a greedy selection mechanism. The winner is retained and it will become a member of next generation. For a minimization problem, the selection process can be described according to the following equation:

(4) $$\begin{array}{c} x_{i}^{⋆}=\left\{\begin{array}{cc} u_{i}, & \text{if }f\left(u_{i}\right)<f\left(x_{i}\right),\\ x_{i}, & \text{otherwise}, \end{array}\right. \end{array}$$

where *f*(*x*) denotes the objective of solution *x* and *x*_*i*_^⋆^ is an offspring corresponding to the target vector *x*_*i*_.

In a word, except for the initialization phase, the aforementioned steps will be repeated in turn until a stopping criterion is reached.

## 3. An Enhanced Differential Evolution Algorithm

### 3.1. Initialization Based on Opposition-Based Learning

Recently, Rahnamayan et al. [12, 13] proposed a new scheme for generating random numbers, called opposition-based learning (OBL), which can effectively make use of random numbers and their opposites. Moreover, the ability of OBL accelerating the optimization, search, or learning process in many soft computing techniques has been reported in the literatures [12, 13]. At first, a state-of-the-art algorithm, named ODE, was proposed by applying the OBL scheme to accelerate DE [13]. After that, the OBL scheme has been successfully used in other evolutionary algorithms such as artificial bee colony algorithm [40], harmony search algorithm [41], particle swarm optimization [42, 43], and teaching learning based algorithm [44]. A comprehensive survey about the OBL scheme can be found in [45].

In order to improve the solution quality of initial population, the OBL scheme is employed to initialize the population individuals of EDE in the work. The initial process can be described as shown in Algorithm 1.

In Algorithm 1, two sets, that is, sets *X* and *OX*, are generated, where *X* = {*x*_1_, *x*_2_,…, *x*_NP_} and *OX* = {*ox*_1_, *ox*_2_,…, *ox*_NP_}. The initial population consists of the top NP individuals chosen from the set *X* ∪ *OX* according to their fitness values.

### 3.2. Multiple Mutation Strategies

A mutation strategy DE/current/1/bin is employed. Namely, the target vector *x*_*i*_ is employed as the base vector in this DE version. That is, a mutant vector *v*_*i*_ will be generated by the following equation:

(5) $$\begin{array}{c} v_{i}=x_{i}+F\cdot \left(x_{a}-x_{b}\right), \end{array}$$

where *i* ∈ {1,2,…, NP} represents the index of current individual, *a* ∈ {1,2,…, NP} and *b* ∈ {1,2,…, NP} are random integers, and *a* ≠ *b* ≠ *i*.

In order to better take advantage of the guiding information of best individual, a new version of DE/best/1/bin, DE/*p*best/1/bin, proposed by Zhang and Sanderson [15], is further employed in the work to speed up the convergence speed of the proposed approach EDE. That is, a mutant vector *v*_*i*_ is produced as follows:

(6) $$\begin{array}{c} v_{i}=x_{best}^{p}+F\cdot \left(x_{a}-x_{b}\right), \end{array}$$

where *p* ∈ {1,2,…, *M*}⊆{1,2,…, NP} is a random number and it denotes the top *p* individuals according to the fitness values of individuals. It should be noted that *p* of DE/*p*best/1/bin in JADE [15] is a proportional number between [0,1].

More specifically, according to the first mutation strategy, it can be seen that new generated mutant vectors will be scattered around the respective target vectors, which can not only keep good population diversity but also avoid the overrandomness of classic mutation strategy DE/rand/1/bin. According to the second mutation strategy DE/*p*best/1/bin, owing to the guidance of one of several better individuals (*x*_best_^*p*^) rather than the only best individual *x*_best_, the used mutation strategy can drive population towards better individuals so as to enhance the convergence speed. In addition, it can also prevent EDE from congregating the vicinity of global best individual to some extent.

In the meantime, a probabilistic parameter *r*_1_ is time varying and designed to control which of the two mutation strategies is to be executed at the mutation step. The parameter *r*_1_ can be described as follows:

(7) $$\begin{array}{c} r_{1}=r_{max}-\frac{\text{FEs}}{\max\text{FEs}}\cdot \left(r_{max}-r_{min}\right), \end{array}$$

where *r*_max_ and *r*_min_ denote the maximum probability value and the minimum probability value, respectively. FEs is an iterative variable. max⁡FEs represents the maximum number of fitness function evaluations.

As a matter of fact, the probability parameter *r*_1_ plays an important role in balancing the exploration ability and the exploitation ability. That is, it is hoped that good population diversity is kept at the beginning of evolution and fast convergence speed is achieved at the end of search.

### 3.3. Perturbation

After repeating all operations (mutation, crossover, and select operations) of differential evolution, a perturbed scheme is conducted over the best individual in order to further trade off the searching ability of the aforementioned solution search equations. During the process, two perturbed equations are introduced and the best individual is perturbed dimension by dimension according to them, which are described by (8) and (9), respectively. One has

(8) $$\begin{array}{c} \mu_{j}=x_{best,n}+\left(2\cdot \mathrm{rand}\left(0,1\right)-1\right)\cdot \left(x_{best,n}-x_{k,n}\right), \end{array}$$

where *j* = 1,2,…, *D*, *μ* = (*μ*_1_, *μ*_2_,…, *μ*_*D*_) is a temporary copy of the best individual, best represents the index of best individual in current population, *k* ∈ {1,2,…, NP}∧*k* ≠ best is a uniform random number, and *n* ∈ {1,2,…, *D*} is also a random number. One has

(9) $$\begin{array}{c} \mu_{j}=x_{best,j}+\left(2\cdot \mathrm{rand}\left(0,1\right)-1\right)\cdot \left(x_{best,n}-x_{k,n}\right), \end{array}$$

where all the notations are the same as those in (8).

From (9), it can be observed that perturbation operation occurs on the current component *j* of best individual, and the differential variation (*x*_best,*n*_ − *x*_*k*,*n*_) acts as perturbed scales. Notice that dimension *n* may be different from *j*, which is helpful to enrich perturbation scales to some extent. That is, it may increase the probability of getting out of local minima trap.

What is more, the term *x*_best,*j*_ of (9) is different from the first term on the right hand side of formulation (8). The reason for (8) introduced is that information between different dimensions of best individual could be shared. Thus, the EDE algorithm could get out of local optimal trap with a larger probability.

Like the aforementioned tradeoff scheme, a probability parameter *r*_2_ is employed. The parameter *r*_2_ is linear time-varying during the evolution process as follows:

(10) $$\begin{array}{c} r_{2}=w_{min}+\frac{\text{FEs}}{\max\text{FEs}}\cdot \left(w_{max}-w_{min}\right), \end{array}$$

where *w*_max_ and *w*_min_ denote the maximum probability value and the minimum probability value, respectively. The rest of these parameters are the same as those in (7).

Concretely speaking, (8) is executed with a probability value *r*_2_, but (9) is executed with a probability value (1 − *r*_2_).

### 3.4. Boundary Constraints Handling Technique

In order to keep solutions subject to boundary constraints, some components of a solution violating the predefined boundary constraints should be repaired. That is, if a parameter value produced by solution search equations exceeds its predefined boundaries, the parameter should be set to an acceptable value. The following repair rule used in the literature [17] is employed in this work:

(11) $$\begin{array}{c} x_{ij}=\left\{\begin{array}{cc} x_{j}^{min}+\mathrm{rand}\left(0,1\right)\cdot \left(x_{j}^{max}-x_{j}^{min}\right), & \text{if }x_{ij}<x_{j}^{min},\\ x_{j}^{max}-\mathrm{rand}\left(0,1\right)\cdot \left(x_{j}^{max}-x_{j}^{min}\right), & \text{if }x_{ij}>x_{j}^{max}. \end{array}\right. \end{array}$$

### 3.5. The Proposed Approach

In order to effectively take use of the guidance information of best individual, mutation strategy DE/best/1/bin is considered. In order to prevent a large number of individuals from clustering around the global best individual, inspired by JADE [15], mutation strategy DE/*p*best/1/bin is actually used. In addition, another mutation strategy DE/current/1/bin is employed to further trade off the exploitation ability of DE/*p*best/1/bin. At the same time, a selective probability *r*_1_ with linear time-varying nature is introduced to decide which mutation strategy works at the mutation phase of DE. Subsequently, a perturbation scheme for the best individual is incorporated into the modified DE version. In short, the pseudocode of EDE can be given in Algorithm 2 based on the above explanation.

## 4. Experimental Study and Discussion

### 4.1. Benchmark Functions and Parameter Settings

To verify the optimization effectiveness of EDE, twenty-five benchmark functions with different characteristics taken from Yao et al. [46], Gong et al. [23], and Gao and Liu [40] are employed here.

These benchmark functions are listed briefly in Table 1, in which *D* designates the dimensionality of test functions. All the functions are scalable and high-dimensional problems. Functions *f*_01_–*f*_05_, *f*_14_, and *f*_15_ are unimodal. Function *f*_06_, that is, the step function, has one minimum and is discontinuous. Function *f*_07_ is a quartic function with noise. Functions *f*_08_–*f*_13_ and *f*_16_–*f*_19_ are difficult multimodal functions where the number of local minima increases exponentially as the dimension of test function increases. In addition, six shifted functions are chosen to evaluate the performance of EDE. Namely, functions *f*_20_–*f*_25_ are shifted functions and *o* = (*o*_1_, *o*_2_,…, *o*_*D*_) representing a shifted vector is generated randomly in the corresponding search range.

In our experimental study, all benchmark functions are tested in 30 dimensions and 100 dimensions. The corresponding maximum number of fitness function evaluations (max⁡FEs) is 15*e*4 and 50*e*4, respectively. Moreover, the other specific parameters of DE and EDE are set as follows.

*DE Settings*. In canonical DE/rand/1/bin, the scale factor *F* is set to 0.5, the parameter of crossover rate Cr is set to 0.9, and the population size SN is 100. It should be noted that the values of three parameters are the same as those of the state-of-the-art algorithm ODE [13].

*EDE Settings*. In our proposed algorithm, the scale factor *F* is set to 0.5. The parameter of crossover rate Cr is set to 0.9. And the population size SN is 20. A few other parameters are set as follows: *r*_max_ = 1, *r*_min_ = 0.1, *w*_max_ = 0.2, *w*_min_ = 0, and *M* = 4.

For the set of experiments tested on 25 benchmark functions, we use the aforementioned parameter settings unless a change is mentioned. Furthermore, each test case is optimized thirty runs independently. Then, experimental results for these well-known problems as well as some comparisons with other famous methods are reported as follows.

### 4.2. Comparison between DE and EDE

**Figure 1:**
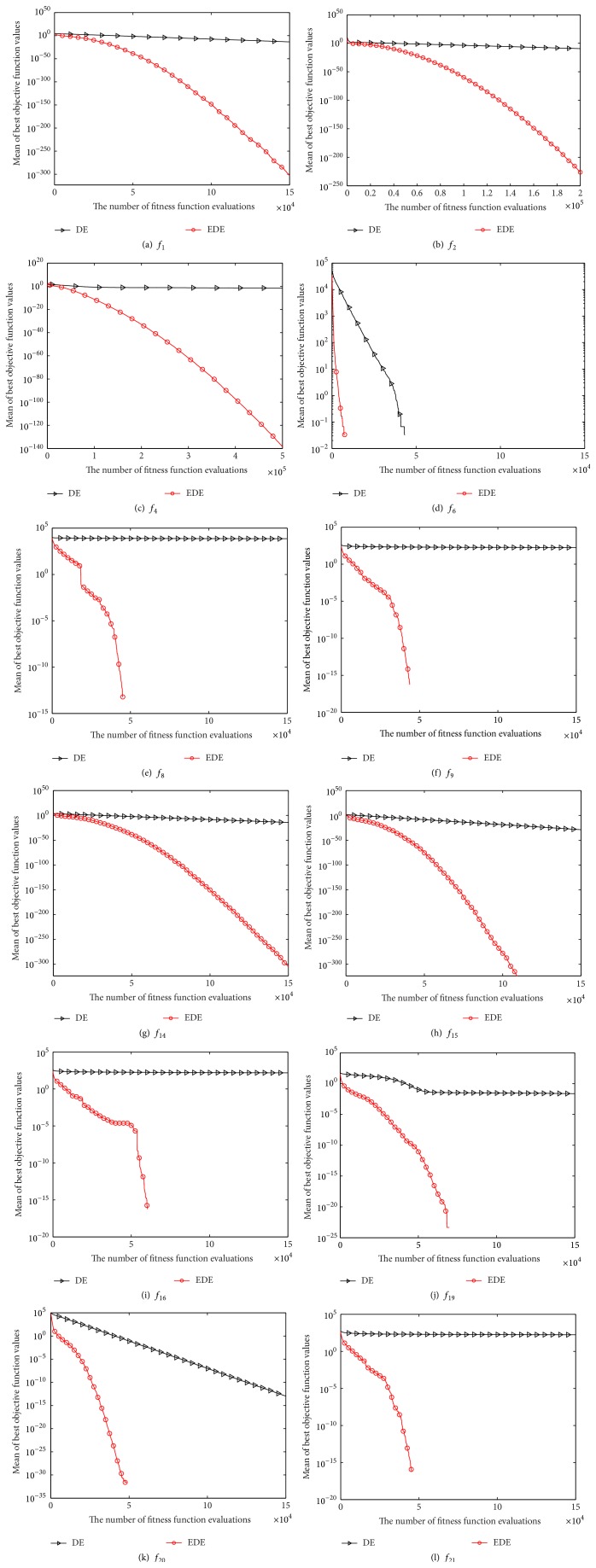
Convergence performance of DE and EDE on the twelve test functions at *D* = 30.

For the purpose of validating the enhancing effectiveness of EDE, EDE is first compared with canonical DE in terms of best, worst, median, mean, and standard deviation (Std.) values of solutions achieved by each algorithm in 30 independent runs. The corresponding results are listed in Table 2. Furthermore, the Wilcoxon rank sum test is conducted to compare the significant difference between DE and EDE at *α* = 0.05 significance level. The related test results are also reported in Table 2. And then, some representatives of convergence curves of DE and EDE are shown in Figure 1 in order to show the convergence speed of EDE more clearly.

From Table 2, it can be seen that EDE is significantly superior to DE in most cases. To be specific, EDE is significantly better than DE on 20 functions, that is, *f*_01_, *f*_02_, *f*_03_, *f*_04_, *f*_05_, *f*_06_, *f*_08_, *f*_09_, *f*_10_, *f*_12_, *f*_13_, *f*_14_, *f*_15_, *f*_16_, *f*_19_, *f*_20_, *f*_21_, *f*_22_, *f*_24_, and *f*_25_, in terms of related Wilcoxon rank sum test results. In addition, for function *f*_07_ with *D* = 30, EDE is still better than DE. For function *f*_07_ with *D* = 100, EDE is equal to DE; actually, the mean result achieved by EDE is slightly better than that of DE. For function *f*_11_ with *D* = 30, EDE is similar to DE. For the function *f*_11_ with *D* = 100, DE outperforms EDE. Nevertheless, the results obtained by EDE are very close to those found by DE. For the functions *f*_17_, *f*_18_ and *f*_23_, DE is better than EDE. And yet, the results obtained by EDE are very close to those found by DE on the functions *f*_17_, *f*_18_ at *D* = 30 and *f*_23_ at *D* = 100.

From Figure 1, it can also be observed that EDE is far better than DE in terms of solutions accuracy and convergence speed on the representative cases.

According to the aforementioned analyses, it can be concluded that EDE is better than or approximately equal to DE on almost all the functions. In other words, multiple mutation strategies and perturbation schemes are beneficial to the performance of EDE.

### 4.3. Comparison between EDE and Other Three DE Variants

In this subsection, EDE is further compared with some representatives of state-of-the-art DE variants, such as SaDE [14], JADE [15], and SaJADE [23]. Here sixteen test functions are used for the comparison. The related comparison results are listed in Table 3. For a fair comparison, except for the proposed algorithm EDE, the rest of the results reported in Table 3 are directly taken from Gong et al. [23].

From Table 3, it can be seen that EDE is obviously better than JADE on twelve functions, that is, *f*_01_, *f*_02_, *f*_04_, *f*_05_, *f*_06_, *f*_08_, *f*_09_, *f*_10_, *f*_12_, *f*_13_, *f*_19_, and *f*_21_. JADE works better than EDE on four functions. Notice that EDE is just slightly inferior to JADE on the three functions *f*_03_, *f*_07_, and *f*_18_. When compared with SaDE, EDE performs better than it does on thirteen functions. And the results found by EDE are very close to those found by SaDE on other two functions *f*_07_ and *f*_18_. When compared with SaJADE, SaJADE is better than EDE on four functions, but the superiority of SaJADE is not obvious on the three functions *f*_05_, *f*_07_, and *f*_18_ except for function *f*_11_. Yet EDE is better than or equal to SaJADE on other twelve functions.

It should be pointed out that the results are summarized as *w*/*t*/*l* in the last line of Table 3, which means that EDE wins in *w* function cases, ties in *t* cases, and loses in *l* cases when compared with its competitor. For JADE, SaDE, and SaJADE, they are 12/0/4, 13/0/3, and 11/1/4, respectively. The results show that EDE is superior to or similar to other three approaches on the majority of benchmark functions.

### 4.4. Comparison among EDE and Two Artificial Bee Colony Algorithms

Artificial bee colony algorithm introduced by Karaboga and Basturk is a relatively new swarm-based optimization algorithm [47]. And it has become a promising technique [48]. Particularly, a modified artificial bee colony algorithm, named MABC, proposed by Gao and Liu [40], is an outstanding representative of many enhanced ABC versions. In order to further demonstrate the superiority of EDE, EDE is compared with standard ABC and MABC on twenty-one functions again. In the experimental study, the maximum number of fitness function evaluations (max⁡FEs) is set to 15*e*4 for all compared algorithms as recommended by Gao and Liu [40].

The further comparison results are given in Table 4. For convenience, besides the data achieved by the EDE algorithm, the rest of the results are gained by Gao and Liu [40] directly.

From Table 4, it is clear that EDE is better than or at least even with ABC on nineteen functions, but ABC only works better than EDE on two functions. EDE is better than or equal to MABC on eighteen functions. MABC also only surpasses EDE on three functions. In addition, the accuracy of solution obtained by EDE is far better than that obtained by ABC on many benchmark functions such as *f*_01_, *f*_02_, *f*_08_, *f*_14_, *f*_15_, and *f*_19_. Meanwhile, the accuracy of solution obtained by EDE is far better than that obtained by MABC on some test functions including *f*_01_, *f*_02_, and *f*_14_. In summary, EDE is superior to both ABC and MABC.

## 5. Conclusion

In order to achieve a better compromise between the exploration ability and the exploitation ability of DE, in this work, an enhanced differential evolution algorithm, called EDE, is presented. In EDE, first, an initialization technique, opposition-based learning initialization, is employed. Next, inspired by JADE [15], a mutation strategy DE/*p*best/1/bin is introduced in EDE. At the same time, a new mutation strategy DE/current/bin/1 is also introduced. That is, there are multiple mutation strategies composed of the two mutation strategies in EDE to better balance the exploration and the exploitation of DE. When performing the EDE algorithm, one of the two mutation strategies is chosen randomly with a linear time-varying scheme. Last, a perturbation scheme for the best individual is presented in order to get out of local minima, where the perturbation scheme is also composed of two solution search equations. Specifically, the best individual is perturbed dimension by dimension in two modes. All these modifications make up the proposed algorithm EDE.

To testify the convergence performance of EDE, twenty-five benchmark functions with different characteristics from literatures are employed. The first experimental results demonstrate that EDE significantly enhances the performance of standard DE in terms of the best, worst, median, mean, and standard deviation (Std.) values of final solutions in most cases. Moreover, other two comparisons also show that EDE performs significantly better than or at least highly competitive with other five well-known algorithms, that is, JADE, SaDE, SaJADE, ABC, and MABC, on the majority of the corresponding benchmark functions. Therefore, it can be concluded that EDE is an efficient method and it may be a good alternative for solving complex numerical optimization problems.

Last but not least, it is desirable to further apply the EDE algorithm to deal with other optimization problems such as the training of neural networks, system parameter identification, and data clustering.

## Figures and Tables

**Algorithm 1:**
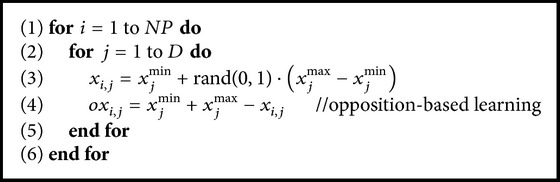
Initialization based on opposition-based learning.

**Algorithm 2:**
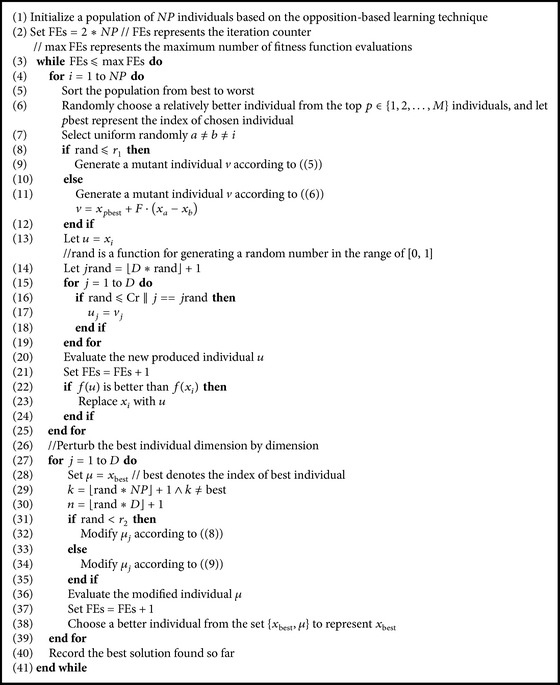
The EDE algorithm.

**Table 1:** Benchmark functions used in experiments.

Test functions	Search space	Optimum
$f_{01}\left(x\right)=\overset{D}{\underset{i=1}{\sum }}x_{i}^{2}$	[−100,100]^*D*^	0
$f_{02}\left(x\right)=\overset{D}{\underset{i=1}{\sum }}\left|x_{i}\right|+\prod_{i=1}^{D}\left|x_{i}\right|$	[−10,10]^*D*^	0
$f_{03}\left(x\right)=\overset{D}{\underset{i=1}{\sum }}{\left(\sum_{j=1}^{i}x_{j}\right)}^{2}$	[−100,100]^*D*^	0
$f_{04}\left(x\right)=\underset{i}{\max}\left\{\left|x_{i}\right|,1⩽i⩽D\right\}$	[−100,100]^*D*^	0
$f_{05}\left(x\right)=\overset{D-1}{\underset{i=1}{\sum }}\left[100{\left(x_{i+1}-x_{i}^{2}\right)}^{2}+{\left(x_{i}-1\right)}^{2}\right]$	[−30,30]^*D*^	0
$f_{06}\left(x\right)=\overset{D}{\underset{i=1}{\sum }}{\left(\left\lfloor x_{i}+0.5\right\rfloor \right)}^{2}$	[−100,100]^*D*^	0
$f_{07}\left(x\right)=\overset{D}{\underset{i=1}{\sum }}ix_{i}^{4}+random[0,1)$	[−1.28,1.28]^*D*^	0
$f_{08}\left(x\right)=-418.98288727243369\times D+\overset{D}{\underset{i=1}{\sum }}\left[-x_{i}\sin\left(\sqrt{\left|x_{i}\right|}\right)\right]$	[−500,500]^*D*^	0
*f*_09_(*x*) = ∑_*i*=1_^*D*^(*x*_*i*_^2^ − 10cos⁡(2*πx*_*i*_) + 10)	[−5.12,5.12]^*D*^	0
$f_{10}\left(x\right)=-20\exp\left(-0.2\sqrt{\frac{1}{D}\sum_{i=1}^{D}x_{i}^{2}}\right)\;-\exp\left(\frac{1}{D}\overset{D}{\underset{i=1}{\sum }}\cos\left(2\pi x_{i}\right)\right)+20+e$	[−32,32]^*D*^	0
$f_{11}\left(x\right)=\frac{1}{4000}\overset{D}{\underset{i=1}{\sum }}x_{i}^{2}-\prod_{i}^{D}\cos\left(\frac{x_{i}}{\sqrt{i}}\right)+1$	[−600,600]^*D*^	0
$f_{12}\left(x\right)=\frac{\pi }{D}\left\{10\sin^{2}\left(\pi y_{1}\right)+\overset{D-1}{\underset{i=1}{\sum }}{\left(y_{i}-1\right)}^{2}\left[1+10\sin^{2}\left(\pi y_{i+1}\right)\right]{\left(y_{n}+1\right)}^{2}\right\}+\overset{D}{\underset{i=1}{\sum }}u\left(x_{i},10,100,4\right)$,	[−50,50]^*D*^	0
where $y_{i}=1+\frac{1}{4}\left(x_{i}+1\right)$, $u\left(x_{i},a,k,m\right)=\left\{\begin{array}{cc} k{\left(x_{i}-a\right)}^{m}, & x_{i}>a;\\ 0, & -a⩽x_{i}⩽a;\\ k{\left(-x_{i}-a\right)}^{m}, & x_{i}<-a. \end{array}\right.$		
$f_{13}\left(x\right)=0.1\left\{{sin}^{2}\left(3\pi x_{1}\right)+\overset{D}{\underset{i=1}{\sum }}{\left(x_{i}-1\right)}^{2}\left[1+\sin^{2}\left(3\pi x_{i}+1\right)\right]+{\left(x_{n}-1\right)}^{2}\left[1+\sin^{2}\left(2\pi x_{n}\right)\right]\right\}+\overset{D}{\underset{i=1}{\sum }}u\left(x_{i},5,100,4\right)$	[−50,50]^*D*^	0
$f_{14}\left(x\right)=\overset{D}{\underset{i=1}{\sum }}ix_{i}^{2}$	[−10,10]^*D*^	0
*f*_15_(*x*) = ∑_*i*=1_^*D*^ *ix*_*i*_^4^	[−1.28,1.28]^*D*^	0
$f_{16}\left(x\right)=\overset{D}{\underset{i=1}{\sum }}\left[y_{i}^{2}-10\cos\left(2\pi y_{i}\right)+10\right]$, $\text{where}\;\;y_{i}=\left\{\begin{array}{cc} x_{i} & \text{if}\;\;\left|x_{i}\right|<0.5,\\ \frac{\text{round}\left(2x_{i}\right)}{2} & \text{else}\;\left|x_{i}\right|⩾0.5. \end{array}\right.$	[−5.12,5.12]^*D*^	0
$f_{17}\left(x\right)=0.5+\frac{\sin^{2}\sqrt{\sum_{i=1}^{D}x_{i}^{2}}-0.5}{{\left(1+0.001\sum_{i=1}^{D}x_{i}^{2}\right)}^{2}}$	[−100,100]^*D*^	0
*f*_18_(*x*) = −cos⁡(2*π*‖*x*‖) + 0.1‖*x*‖ + 1, where $\left\|x\right\|=\sqrt{\sum_{i=1}^{D}x_{i}^{2}}$	[−100,100]^*D*^	0
$f_{19}\left(x\right)=\overset{D}{\underset{i=1}{\sum }}\left|x_{i}\sin\left(x_{i}\right)+0.1x_{i}\right|$	[−10,10]^*D*^	0
$f_{20}\left(x\right)=\overset{D}{\underset{i=1}{\sum }}z_{i}^{2}$ , *z* = *x* − *o*	[−100,100]^*D*^	0
$f_{21}\left(x\right)=\overset{D}{\underset{i=1}{\sum }}\left(z_{i}^{2}-10\cos\left(2\pi z_{i}\right)+10\right)$, *z* = *x* − *o*	[−5,5]^*D*^	0
$f_{22}\left(x\right)=-20\exp\left(-0.2\sqrt{\frac{1}{D}\sum_{i=1}^{D}z_{i}^{2}}\right)-\exp\left(\frac{1}{D}\overset{D}{\underset{i=1}{\sum }}\cos\left(2\pi z_{i}\right)\right)+20+e$, *z* = *x* − *o*	[−32,32]^*D*^	0
$f_{23}\left(x\right)=\frac{1}{4000}\sum_{i=1}^{D}z_{i}^{2}-\prod_{i}^{D}\cos\left(\frac{z_{i}}{\sqrt{i}}\right)+1$, *z* = *x* − *o*	[−600,600]^*D*^	0
$f_{24}\left(x\right)=\overset{D}{\underset{i=1}{\sum }}{\left(\overset{i}{\underset{j=1}{\sum }}z_{j}\right)}^{2}$, *z* = *x* − *o*	[−100,100]^*D*^	0
$f_{25}\left(x\right)=\overset{D-1}{\underset{i=1}{\sum }}\left(100{\left(z_{i+1}-z_{i}^{2}\right)}^{2}+{\left(z_{i}-1\right)}^{2}\right)$, *z* = *x* − *o* + 1	[−100,100]^*D*^	0

**Table 2:** Best, worst, median, mean, and standard deviation values achieved by DE and EDE through 30 independent runs.

Number	Dim.	max⁡FEs	Methods	Best	Worst	Median	Mean	Std.	Sig.
*f*_01_	30	15*e*4	DE	1.48*e* − 014	1.00*e* − 013	3.38*e* − 014	3.81*e* − 014	1.87*e* − 014	†
			EDE	0.00*e* − 000	1.13*e* − 302	1.12*e* − 315	4.19*e* − 304	0.00*e* − 000	
	100	50*e*4	DE	1.50*e* − 018	9.93*e* − 017	7.79*e* − 018	1.29*e* − 017	1.86*e* − 017	†
			EDE	0.00*e* − 000	0.00*e* − 000	0.00*e* − 000	0.00*e* − 000	0.00*e* − 000	

*f*_02_	30	20*e*4	DE	1.40*e* − 010	9.17*e* − 010	3.57*e* − 010	3.95*e* − 010	1.93*e* − 010	†
			EDE	6.18*e* − 234	1.00*e* − 225	1.05*e* − 229	4.65*e* − 227	0.00*e* − 000	
	100	50*e*4	DE	1.39*e* − 010	6.55*e* − 010	3.61*e* − 010	3.71*e* − 010	1.30*e* − 010	†
			EDE	8.41*e* − 229	2.02*e* − 221	5.42*e* − 225	7.34*e* − 223	0.00*e* − 000	

*f*_03_	30	50*e*4	DE	8.22*e* − 013	1.93*e* − 010	2.21*e* − 011	4.23*e* − 011	4.84*e* − 011	†
			EDE	7.63*e* − 085	7.80*e* − 079	3.78*e* − 081	8.01*e* − 080	1.71*e* − 079	
	100	50*e*4	DE	5.27*e* + 003	2.23*e* + 004	9.65*e* + 003	1.01*e* + 004	3.62*e* + 003	†
			EDE	3.64*e* − 004	1.18*e* − 002	2.50*e* − 003	3.70*e* − 003	3.20*e* − 003	

*f*_04_	30	50*e*4	DE	4.40*e* − 012	2.50*e* − 001	1.00*e* − 003	3.15*e* − 002	6.32*e* − 002	†
			EDE	1.43*e* − 144	2.81*e* − 138	2.95*e* − 140	2.79*e* − 139	6.39*e* − 139	
	100	50*e*4	DE	1.01*e* + 001	2.67*e* + 001	1.97*e* + 001	1.97*e* + 001	3.30*e* − 000	†
			EDE	3.61*e* − 027	7.99*e* − 025	3.92*e* − 026	1.02*e* − 025	1.59*e* − 025	

*f*_05_	30	15*e*4	DE	1.41*e* + 001	1.84*e* + 001	1.70*e* + 001	1.68*e* + 001	1.06*e* − 000	†
			EDE	1.52*e* − 024	2.43*e* − 001	4.03*e* − 015	8.50*e* − 003	4.43*e* − 002	
		50*e*4	DE	3.47*e* − 016	3.98*e* − 000	1.99*e* − 013	1.32*e* − 001	7.27*e* − 001	†
			EDE	0.00*e* − 000	7.28*e* − 027	2.17*e* − 029	4.42*e* − 028	1.38*e* − 027	
	100	50*e*4	DE	7.79*e* + 001	1.96*e* + 002	1.41*e* + 002	1.33*e* + 002	3.63*e* + 001	†
			EDE	1.19*e* − 004	1.61*e* + 002	7.44*e* + 001	5.31*e* + 001	4.70*e* + 001	

*f*_06_	30	8*e*3	DE	1.79*e* + 003	5.38*e* + 003	4.07*e* + 003	3.90*e* + 003	8.52*e* + 002	†
			EDE	0.00*e* − 000	0.00*e* − 000	0.00*e* − 000	0.00*e* − 000	0.00*e* − 000	
		15*e*4	DE	0.00*e* − 000	0.00*e* − 000	0.00*e* − 000	0.00*e* − 000	0.00*e* − 000	
			EDE	0.00*e* − 000	0.00*e* − 000	0.00*e* − 000	0.00*e* − 000	0.00*e* − 000	
	100	5*e*4	DE	4.27*e* + 002	1.06*e* + 003	6.83*e* + 002	6.81*e* + 002	1.65*e* + 002	†
			EDE	0.00*e* − 000	0.00*e* − 000	0.00*e* − 000	0.00*e* − 000	0.00*e* − 000	
		50*e*4	DE	0.00*e* − 000	0.00*e* − 000	0.00*e* − 000	0.00*e* − 000	0.00*e* − 000	
			EDE	0.00*e* − 000	0.00*e* − 000	0.00*e* − 000	0.00*e* − 000	0.00*e* − 000	

*f*_07_	30	30*e*4	DE	2.50*e* − 003	8.10*e* − 003	4.40*e* − 003	4.70*e* − 003	1.40*e* − 003	†
			EDE	7.02*e* − 004	3.80*e* − 003	2.30*e* − 003	2.30*e* − 003	8.87*e* − 004	
	100	50*e*4	DE	1.80*e* − 002	9.09*e* − 002	2.87*e* − 002	3.25*e* − 002	1.34*e* − 003	≈
			EDE	2.29*e* − 002	4.25*e* − 002	3.03*e* − 002	3.05*e* − 002	4.90*e* − 003	

*f*_08_	30	15*e*4	DE	6.56*e* + 003	7.72*e* + 003	7.26*e* + 003	7.26*e* + 003	2.91*e* + 002	†
			EDE	0.00*e* − 000	0.00*e* − 000	0.00*e* − 000	0.00*e* − 000	0.00*e* − 000	
	100	50*e*4	DE	2.56*e* + 004	3.30*e* + 004	2.89*e* + 004	2.93*e* + 004	1.84*e* + 003	†
			EDE	9.45*e* − 011	9.45*e* − 011	9.45*e* − 011	9.45*e* − 011	0.00*e* − 000	

*f*_09_	30	15*e*4	DE	1.46*e* + 002	1.94*e* + 002	1.77*e* + 002	1.74*e* + 002	1.34*e* + 001	†
			EDE	0.00*e* − 000	0.00*e* − 000	0.00*e* − 000	0.00*e* − 000	0.00*e* − 000	
	100	50*e*4	DE	1.91*e* + 002	6.65*e* + 002	5.70*e* + 002	5.49*e* + 002	1.03*e* + 002	†
			EDE	0.00*e* − 000	0.00*e* − 000	0.00*e* − 000	0.00*e* − 000	0.00*e* − 000	

*f*_10_	30	15*e*4	DE	2.82*e* − 008	2.07*e* − 007	5.86*e* − 008	7.10*e* − 008	3.55*e* − 008	†
			EDE	4.44*e* − 015	4.44*e* − 015	4.44*e* − 015	4.44*e* − 015	0.00*e* − 000	
	100	50*e*4	DE	2.06*e* − 010	2.79*e* − 009	5.90*e* − 010	6.86*e* − 010	4.79*e* − 010	†
			EDE	4.44*e* − 015	7.99*e* − 015	7.99*e* − 015	7.40*e* − 015	1.34*e* − 015	

*f*_11_	30	15*e*4	DE	1.16*e* − 014	7.40*e* − 003	1.11*e* − 013	4.93*e* − 004	1.90*e* − 003	≈
			EDE	0.00*e* − 000	7.34*e* − 002	1.60*e* − 002	2.09*e* − 002	2.19*e* − 002	
	100	50*e*4	DE	0.00*e* − 000	5.37*e* − 002	0.00*e* − 000	3.20*e* − 003	1.01*e* − 002	-
			EDE	0.00*e* − 000	6.11*e* − 002	0.00*e* − 000	1.05*e* − 002	1.76*e* − 002	

*f*_12_	30	15*e*4	DE	8.61*e* − 016	2.17*e* − 014	3.90*e* − 015	5.28*e* − 015	4.65*e* − 015	†
			EDE	1.57*e* − 032	1.57*e* − 032	1.57*e* − 032	1.57*e* − 032	5.56*e* − 048	
	100	50*e*4	DE	5.48*e* − 019	1.55*e* − 001	2.46*e* − 016	8.30*e* − 003	2.94*e* − 002	†
			EDE	4.71*e* − 033	4.71*e* − 033	4.71*e* − 033	4.71*e* − 033	1.39*e* − 048	

*f*_13_	30	15*e*4	DE	8.68*e* − 015	1.06*e* − 013	2.61*e* − 014	3.55*e* − 014	2.46*e* − 014	†
			EDE	1.34*e* − 032	1.34*e* − 032	1.34*e* − 032	1.34*e* − 032	5.56*e* − 048	
	100	50*e*4	DE	2.74*e* − 017	2.75*e* + 001	9.82*e* − 000	1.02*e* + 001	6.88*e* − 000	†
			EDE	1.34*e* − 032	1.34*e* − 032	1.34*e* − 032	1.34*e* − 032	5.56*e* − 048	

*f*_14_	30	15*e*4	DE	7.58*e* − 016	1.86*e* − 014	5.13*e* − 015	5.71*e* − 015	4.17*e* − 015	†
			EDE	0.00*e* − 000	6.48*e* − 304	1.00*e* − 313	2.19*e* − 305	0.00*e* − 000	
	100	50*e*4	DE	2.21*e* − 019	2.62*e* − 017	3.41*e* − 018	4.70*e* − 018	4.93*e* − 018	†
			EDE	0.00*e* − 000	0.00*e* − 000	0.00*e* − 000	0.00*e* − 000	0.00*e* − 000	

*f*_15_	30	15*e*4	DE	5.46*e* − 031	1.13*e* − 028	5.45*e* − 030	1.53*e* − 029	2.50*e* − 029	†
			EDE	0.00*e* − 000	0.00*e* − 000	0.00*e* − 000	0.00*e* − 000	0.00*e* − 000	
	100	50*e*4	DE	1.58*e* − 027	3.78*e* − 024	3.00*e* − 026	1.97*e* − 025	6.84*e* − 025	†
			EDE	0.00*e* − 000	0.00*e* − 000	0.00*e* − 000	0.00*e* − 000	0.00*e* − 000	

*f*_16_	30	15*e*4	DE	1.18*e* + 002	1.70*e* + 002	1.52*e* + 002	1.51*e* + 002	1.37*e* + 001	†
			EDE	0.00*e* − 000	0.00*e* − 000	0.00*e* − 000	0.00*e* − 000	0.00*e* − 000	
	100	50*e*4	DE	3.64*e* + 002	7.47*e* + 002	6.07*e* + 002	6.05*e* + 002	9.01*e* + 001	†
			EDE	0.00*e* − 000	0.00*e* − 000	0.00*e* − 000	0.00*e* − 000	0.00*e* − 000	

*f*_17_	30	15*e*4	DE	3.72*e* − 002	3.72*e* − 002	3.72*e* − 002	3.72*e* − 002	5.43*e* − 007	-
			EDE	7.82*e* − 002	2.72*e* − 001	1.78*e* − 001	1.63*e* − 001	5.62*e* − 002	
	100	50*e*4	DE	7.82*e* − 002	1.41*e* − 001	7.82*e* − 002	9.29*e* − 002	2.29*e* − 002	-
			EDE	4.79*e* − 001	4.94*e* − 001	4.90*e* − 001	4.89*e* − 001	4.50*e* − 003	

*f*_18_	30	15*e*4	DE	1.12*e* − 001	2.00*e* − 001	1.99*e* − 001	1.97*e* − 001	1.60*e* − 002	-
			EDE	2.99*e* − 001	6.99*e* − 001	4.99*e* − 001	5.03*e* − 001	1.09*e* − 001	
	100	50*e*4	DE	2.99*e* − 001	3.99*e* − 001	3.07*e* − 001	3.39*e* − 001	4.65*e* − 002	-
			EDE	2.09*e* − 000	3.39*e* − 000	2.79*e* − 000	2.77*e* − 000	3.31*e* − 001	

*f*_19_	30	15*e*4	DE	1.85*e* − 002	2.87*e* − 002	2.39*e* − 002	2.38*e* − 002	2.70*e* − 002	†
			EDE	0.00*e* − 000	0.00*e* − 000	0.00*e* − 000	0.00*e* − 000	0.00*e* − 000	
	100	50*e*4	DE	2.74*e* − 010	3.27*e* − 008	2.84*e* − 009	4.88*e* − 009	6.52*e* − 009	†
			EDE	0.00*e* − 000	0.00*e* − 000	0.00*e* − 000	0.00*e* − 000	0.00*e* − 000	

*f*_20_	30	15*e*4	DE	1.55*e* − 014	5.02*e* − 013	7.96*e* − 014	1.07*e* − 013	1.07*e* − 013	†
			EDE	0.00*e* − 000	0.00*e* − 000	0.00*e* − 000	0.00*e* − 000	0.00*e* − 000	
	100	50*e*4	DE	1.51*e* − 017	1.97*e* − 016	4.43*e* − 017	5.78*e* − 017	3.86*e* − 017	†
			EDE	0.00*e* − 000	4.93*e* − 032	0.00*e* − 000	6.57*e* − 033	1.70*e* − 032	

*f*_21_	30	15*e*4	DE	1.49*e* + 002	1.95*e* + 002	1.79*e* + 002	1.77*e* + 002	1.18*e* + 001	†
			EDE	0.00*e* − 000	0.00*e* − 000	0.00*e* − 000	0.00*e* − 000	0.00*e* − 000	
	100	50*e*4	DE	2.56*e* + 002	7.10*e* + 002	6.09*e* + 002	5.90*e* + 002	1.07*e* + 002	†
			EDE	0.00*e* − 000	0.00*e* − 000	0.00*e* − 000	0.00*e* − 000	0.00*e* − 000	

*f*_22_	30	15*e*4	DE	3.20*e* − 008	2.01*e* − 007	6.56*e* − 008	7.75*e* − 008	3.80*e* − 008	†
			EDE	4.44*e* − 015	7.99*e* − 015	7.99*e* − 015	7.40*e* − 015	1.34*e* − 015	
	100	50*e*4	DE	3.52*e* − 010	1.42*e* − 009	7.04*e* − 010	7.54*e* − 010	2.85*e* − 010	†
			EDE	7.99*e* − 015	7.99*e* − 015	7.99*e* − 015	7.99*e* − 015	0.00*e* − 000	

*f*_23_	30	15*e*4	DE	1.17*e* − 013	2.35*e* − 012	5.77*e* − 013	7.79*e* − 013	6.66*e* − 013	-
			EDE	0.00*e* − 000	1.29*e* − 001	9.90*e* − 003	2.10*e* − 002	2.70*e* − 002	
	100	50*e*4	DE	0.00*e* − 000	9.90*e* − 003	0.00*e* − 000	9.03*e* − 004	2.80*e* − 003	-
			EDE	0.00*e* − 000	3.94*e* − 002	7.40*e* − 003	8.50*e* − 003	1.05*e* − 002	

*f*_24_	30	15*e*4	DE	8.85*e* − 001	5.87*e* − 000	1.77*e* − 000	2.29*e* − 000	1.30*e* − 000	†
			EDE	1.62*e* − 023	2.05*e* − 019	5.25*e* − 021	2.05*e* − 020	4.02*e* − 020	
	100	50*e*4	DE	1.93*e* + 004	5.73*e* + 004	2.76*e* + 004	2.90*e* + 004	6.85*e* + 003	†
			EDE	6.45*e* − 004	2.96*e* − 003	6.20*e* − 003	7.40*e* − 003	6.60*e* − 003	

*f*_25_	30	15*e*4	DE	1.64*e* + 001	7.61*e* + 001	1.84*e* + 001	2.03*e* + 001	1.05*e* + 001	†
			EDE	4.23*e* − 015	7.36*e* − 001	2.72*e* − 007	6.93*e* − 002	1.79*e* − 001	
	100	50*e*4	DE	7.78*e* + 001	3.01*e* + 002	1.43*e* + 002	1.54*e* + 002	5.62*e* + 001	†
			EDE	2.62*e* − 005	1.72*e* + 002	7.59*e* + 001	6.08*e* + 001	4.64*e* + 001	

† indicates that EDE is better than its competitor by the Wilcoxon rank sum test at *α* = 0.05.

- means that EDE is worse than its competitor.

≈ means that there is no significant difference between DE and EDE.

**Table 3:** Performance comparison between EDE and other three DEs over 30 independent runs for the 16 test functions at *D* = 30, where “*w*/*t*/*l*” means that EDE wins in *w* functions, ties in *t* functions, and loses in *l* functions, compared with its competitors.

Number	max⁡FEs	JADE-*w*	SaDE	SaJADE	EDE
*f*_01_	15*e*4	2.69*e* − 56 (1.41*e* − 55)^†^	3.42*e* − 37 (3.63*e* − 37)^†^	1.10*e* − 79 (7.52*e* − 79)^†^	4.19**e** − 304 (0.00**e** − 000)
*f*_02_	20*e*4	3.18*e* − 25 (2.05*e* − 24)^†^	3.51*e* − 25 (2.74*e* − 25)^†^	1.35*e* − 47 (7.53*e* − 47)^†^	4.65**e** − 227 (0.00**e** − 000)
*f*_03_	50*e*4	6.11**e** − 81 (1.62**e** − 80)^‡^	1.54*e* − 14 (4.56*e* − 14)^†^	1.77*e* − 77 (3.39*e* − 77)^†^	8.01*e* − 080 (1.71*e* − 079)
*f*_04_	50*e*4	5.29*e* − 14 (2.05*e* − 14)^†^	6.39*e* − 27 (8.27*e* − 27)^†^	1.26*e* − 19(1.35*e* − 19)^†^	2.79**e** − 139 (6.39**e** − 139)
*f*_05_	50*e*4	1.59*e* − 01(7.89*e* − 01)^†^	7.98*e* − 02 (5.64*e* − 01)^†^	1.60**e** − 30 (6.32**e** − 30)^‡^	4.42*e* − 028 (1.38*e* − 027)
*f*_06_	1*e*4	5.62*e* − 00 (1.87*e* − 00)^†^	5.07*e* + 01 (1.34*e* + 01)^†^	0.00**e** − 00 (0.00**e** − 00)^≈^	0.00**e** − 000 (0.00**e** − 000)
*f*_07_	30*e*4	6.14*e* − 04 (2.55*e* − 04)^‡^	2.06*e* − 03 (5.21*e* − 04)^‡^	4.10**e** − 04 (1.48**e** − 04)^‡^	2.30*e* − 003 (8.87*e* − 004)
*f*_08_	10*e*4	2.62*e* − 04 (3.59*e* − 04)^†^	1.13*e* − 08 (1.08*e* − 08)^†^	6.83*e* − 07 (2.70*e* − 06)^†^	0.00**e** − 000 (0.00**e** − 000)
*f*_09_	10*e*4	1.33*e* − 01 (9.74*e* − 02)^†^	2.43*e* − 00 (1.60*e* − 00)^†^	1.54*e* − 01 (2.25*e* − 01)^†^	0.00**e** − 000 (0.00**e** − 000)
*f*_10_	5*e*4	3.35*e* − 09(2.84*e* − 09)^†^	3.81*e* − 06 (8.26*e* − 07)^†^	1.12*e* − 12 (1.07*e* − 12)^†^	5.03**e** − 015 (1.34**e** − 015)
*f*_11_	5*e*4	1.57*e* − 08 (1.09*e* − 07)^‡^	2.52*e* − 09 (1.24*e* − 08)^‡^	0.00**e** − 00 (0.00**e** − 00)^‡^	2.31*e* − 002 (2.44*e* − 002)
*f*_12_	5*e*4	1.67*e* − 15 (1.02*e* − 14)^†^	8.25*e* − 12 (5.12*e* − 12)^†^	2.10*e* − 23 (6.89*e* − 23)^†^	1.57**e** − 032 (5.56**e** − 048)
*f*_13_	5*e*4	1.87*e* − 10 (1.09*e* − 09)^†^	1.93*e* − 09 (1.53*e* − 09)^†^	3.83*e* − 21(1.56*e* − 20)^†^	1.35**e** − 032 (5.56**e** − 048)
*f*_18_	30*e*4	2.00*e* − 01 (1.63*e* − 02)^‡^	1.56**e** − 01 (5.01**e** − 02)^‡^	1.76*e* − 01 (4.28*e* − 02)^‡^	4.33*e* − 001 (8.02*e* − 002)
*f*_19_	30*e*4	1.87*e* − 10 (2.09*e* − 09)^†^	1.93*e* − 09 (1.53*e* − 09)^†^	3.83*e* − 21 (1.56*e* − 20)^†^	0.00**e** − 000 (0.00**e** − 000)
*f*_21_	10*e*4	1.35*e* − 00 (6.08*e* − 01)^†^	1.46*e* − 00 (1.02*e* − 00)^†^	1.13*e* − 01 (1.60*e* − 01)^†^	0.00**e** − 000 (0.00**e** − 000)

*w*/*t*/*l*		12/0/4	13/0/3	11/1/4	—

† indicates that EDE is better than its competitor.

‡ means that EDE is worse than its competitor.

≈ means that the performance of the corresponding algorithm is even with that of EDE.

Bold entities mean the best results.

**Table 4:** Comparison between EDE and other two ABCs over 30 independent runs on the 21 test functions with *D* = 30 in terms of mean and standard deviation.

Number	max⁡FEs	ABC	MABC	EDE
*f*_01_	15*e*4	5.21*e* − 010 (2.46*e* − 010)	9.43*e* − 032 (6.67*e* − 032)	4.19**e** − 304 (0.00**e** − 000)
*f*_02_	15*e*4	1.83*e* − 006 (4.80*e* − 007)	2.40*e* − 017 (9.02*e* − 018)	1.24**e** − 169 (0.00**e** − 000)
*f*_04_	15*e*4	1.80*e* + 001 (2.25*e* − 000)	1.02*e* + 001 (1.49*e* − 000)	1.65**e** − 041 (2.79**e** − 041)
*f*_05_	15*e*4	4.23*e* − 001 (4.34*e* − 001)	6.11*e* − 001 (4.55*e* − 001)	8.50**e** − 003 (4.43**e** − 002)
*f*_06_	15*e*4	0.00**e** − 000 (0.00**e** − 000)	0.00**e** − 000 (0.00**e** − 000)	0.00**e** − 000 (0.00**e** − 000)
*f*_07_	15*e*4	8.74*e* − 002 (1.77*e* − 002)	3.71*e* − 002 (8.53*e* − 003)	4.90**e** − 003 (2.40**e** − 003)
*f*_08_	15*e*4	8.86*e* + 001 (8.62*e* + 001)	−1.21**e** − 013 (4.53**e** − 013)	0.00*e* − 000 (0.00*e* − 000)^a^
*f*_09_	15*e*4	4.81*e* − 003 (2.57*e* − 002)	0.00**e** − 000 (0.00**e** − 000)	0.00**e** − 000 (0.00**e** − 000)
*f*_10_	15*e*4	4.83*e* − 006 (2.12*e* − 006)	4.13*e* − 014 (2.17*e* − 015)	4.44**e** − 015 (0.00**e** − 000)
*f*_11_	15*e*4	1.61*e* − 008 (3.99*e* − 008)	0.00**e** − 000 (0.00**e** − 000)	2.09*e* − 002 (2.19*e* − 002)
*f*_12_	15*e*4	1.39*e* − 011 (3.82*e* − 012)	1.90*e* − 032 (3.70*e* − 033)	1.57**e** − 032(5.56**e** − 048)
*f*_13_	15*e*4	1.06*e* − 009 (4.24*e* − 010)	2.23*e* − 031 (1.46*e* − 031)	1.34**e** − 032(5.56**e** − 048)
*f*_14_	15*e*4	2.22*e* − 011 (1.14*e* − 011)	2.10*e* − 032 (1.56*e* − 032)	2.19**e** − 305 (0.00**e** − 000)
*f*_15_	15*e*4	5.51*e* − 029 (6.70*e* − 029)	1.45*e* − 067 (2.28*e* − 067)	0.00**e** − 000 (0.00**e** − 000)
*f*_16_	15*e*4	1.12*e* − 001 (2.97*e* − 001)	0.00**e** − 000 (0.00**e** − 000)	0.00**e** − 000 (0.00**e** − 000)
*f*_17_	15*e*4	4.41*e* − 001 (1.81*e* − 002)	2.95*e* − 001 (3.17*e* − 002)	1.63**e** − 001 (5.62**e** − 002)
*f*_19_	15*e*4	7.66*e* − 005 (2.76*e* − 005)	1.58*e* − 016 (2.48*e* − 016)	0.00**e** − 000 (0.00**e** − 000)
*f*_20_	15*e*4	1.55*e* − 009 (5.54*e* − 010)	0.00**e** − 000 (0.00**e** − 000)	0.00**e** − 000 (0.00**e** − 000)
*f*_21_	15*e*4	1.49*e* − 001 (3.55*e* − 001)	0.00**e** − 000 (0.00**e** − 000)	0.00**e** − 000 (0.00**e** − 000)
*f*_22_	15*e*4	9.73*e* − 005 (5.69*e* − 005)	4.92*e* − 014 (5.31*e* − 015)	7.40**e** − 015 (1.34**e** − 015)
*f*_23_	15*e*4	4.93*e* − 004 (2.25*e* − 003)	0.00**e** − 000 (0.00**e** − 000)	2.10*e* − 002 (2.70*e* − 002)

*w*/*t*/*l*		18/1/2	13/5/3	—

Bold entities mean the best results.

Here “a” means that the results obtained by EDE are set to zero on the function *f*_8_ when the results are less than 1*e* − 308. This is the reason that the coefficient −418.98288727243369 with low precision in function *f*_8_ may result in the negative results. As a matter of fact, the results should be zero.
